# AI-driven nano-repair: the future of skin barrier restoration in atopic dermatitis

**DOI:** 10.1097/MS9.0000000000003517

**Published:** 2025-06-23

**Authors:** Muhammad Talha, Maliha Khalid, Laiba Shamim, Aminath Waafira

**Affiliations:** a Department of Medicine, King Edward Medical University, Lahore, Pakistan; b Department of Medicine, Jinnah Sindh Medical University, Karachi, Pakistan; c Department of Medicine, The Maldives National University, Malé, Maldives

*To the Editor,*

Atopic dermatitis (AD) is a long-term allergic skin condition affecting almost 129 million individuals around the world as of 2021. This number is expected to keep rising primarily because of a growing and aging population. It is symptomatic of the disease burden, especially for high-income countries, because the environmental factors and urbanization were thought to play a virulent role in the additional prevalence and disability-adjusted life years (DALY) accrued^[^1^]^. Despite the prevalence remaining static in terms of age standardization, the absolute numbers of individuals affected by the disease have risen, which highlights the dire need for new drug delivery treatments. This letter introduces AI-driven nanorepair systems as a transformative solution for AD management, aligning with the Transparency In The reporting of Artificial Intelligence (TITAN) Guidelines to ensure ethical and transparent AI use in medical research^[^2^]^.

Advances in artificial intelligence (AI) have enabled the creation of nanomaterials specifically designed to repair the damaged skin barrier in individuals with atopic dermatitis (AD). AI-aided algorithms optimize the nanomaterial properties needed to improve skin penetration, ceramide synthesis, and immunomodulation by studying complex datasets that comprise molecular structures, patient genetics, and clinical phenotypes. For example, AI-designed lipid-based nanoparticles have proved to be quite effective as their entry into the stratum corneum was constructed. In addition to damage to the aforementioned bovine epidermis, AD pathophysiology is also caused^[^3^]^. Further, these nanosystems, consisting of reactive oxygen species scavengers and immune modulators, had substantial improvement of symptoms for preclinical models^[^4^]^. Personalized treatments for those who will 1 day experience the benefit of these types of systems, designed through the principles of AI, will ultimately surpass even the best of what current topical treatments can provide.

Emerging clinical evidence is building in favor of the integration of AI in AD management. For example, a 2025 study of an AI-assisted decision-making system demonstrated a 40% improvement in disease severity scores by dynamically modifying treatments based on real-time data from patients^[^5^]^. Also, AI wearable or wearable-integrated systems have reduced 28% nocturnal scratching, which would be a major symptom contributing to disease exacerbation^[^6^]^. AI advances will help not only in the design of nanomaterials but also in patients’ monitoring and adherence, which might help improve long-term outcomes.

Designing for cost-effective scales of manufacturing, regulatory approval, and equitable access remains a major challenge in the present climate. Ethical issues concerning patient data privacy and AI transparency must also be satisfactorily addressed to instill the required confidence and buy-in for mainstream adoption. Interdisciplinary collaboration among dermatologists, AI specialists, and policymakers must continue so that safety standards can be established and AI-based nano-repair systems integrated into everyday clinical practice.

This manuscript discusses potentially revolutionary AI-advanced nano-repair systems to treat atopic dermatitis (AD) by restoring skin-barrier function and facilitating individualized therapy. Different from traditional therapies, it uses AI to enhance nanomaterial design and patient outcomes. In summary: AI makes possible the better design of nanomaterials; real-time monitoring helps compliance and symptom control; and respect for ethical standards provides for the responsible use of AI in dermatology.

To conclude, AI-designed nanomaterials greatly change therapy methodology in atopic dermatitis because they can directly treat the damaged skin barrier without increasing the complexity of the therapy regimen by customizing treatment. Increased investments will be critical to realizing the promising prospects of these technologies in clinical trials, regulatory frameworks, and public health strategies. Such efforts will significantly transform the lives of millions who suffer from atopic dermatitis all over the world.

## Data Availability

No data were generated for this manuscript.
